# On the challenge of estimating diphoton backgrounds at large invariant mass

**DOI:** 10.1140/epjc/s10052-017-4687-y

**Published:** 2017-02-24

**Authors:** J. F. Kamenik, G. Perez, M. Schlaffer, A. Weiler

**Affiliations:** 1 Jožef Stefan Institute, Jamova 39, 1000 Ljubljana, Slovenia; 2 Faculty of Mathematics and Physics, University of Ljubljana, Jadranska 19, 1000 Ljubljana, Slovenia; 3 Department of Particle Physics and Astrophysics, Weizmann Institute of Science, 7610001 Rehovot, Israel; 4 Physik Department T75, Technische Universität München, James-Franck-Strasse 1, 85748 Garching, Germany

## Abstract

We examine, using the analyses of the 750 GeV diphoton resonance as a case study, the methodology for estimating the dominant backgrounds to diphoton resonance searches. We show that close to the high energy tails of the distributions, where background estimates rely on functional extrapolations or Monte Carlo predictions, large uncertainties are introduced, in particular by the challenging photon–jet background. Analyses with loose photon and low photon $p_T$ cuts and those susceptible to high photon rapidity regions are especially affected. Given that diphoton-based searches beyond 1 TeV are highly motivated as discovery modes, these considerations are relevant for future analyses. We first consider a physics-driven deformation of the photon–jet spectrum by next-to-leading order effects and a phase space dependent fake rate and show that this reduces the local significance of the excess. Using a simple but more general ansatz, we demonstrate that the originally reported local significances of the 750 GeV excess could have been overestimated by more than one standard deviation. We furthermore cross-check our analysis by comparing fit results based on the 2015 and 2016 LHC data sets. Finally we employ our methodology on the available 13 TeV LHC data set assessing the systematics involved in the current diphoton searches beyond the TeV region.

## Introduction

Searches for new physics at the energy frontier often look for new phenomena at the edge of distributions. In this kinematical region the knowledge of the standard model (SM) background is typically limited and the challenge is to look for a new resonance where only partial knowledge on the SM background is available. In this paper we focus in particular on new physics probes based on the high diphoton invariant mass spectrum. We examine, using the analyses of the 750 GeV diphoton resonance as a case study, the strategy currently used by the experimental collaborations in estimating the dominant SM backgrounds. We employ our methodology on the 13 TeV LHC data set to asses the systematics involved in the current diphoton searches beyond the TeV region.

In their 2015 data sets, both ATLAS and CMS observed an excess in the diphoton spectrum near $m_{\gamma \gamma }=750\,\,\text {GeV}\,.$ The relevant details of the ATLAS and CMS analyses are described in [1, 2]. At face value the local significances for a broad resonance were given by

1.1 $$\begin{aligned} \begin{array}{ll} p_{\text {ATLAS}}=4 \times 10^{-5}, \\ \sigma _{\text {ATLAS}} = 3.9, \end{array} \quad \begin{array}{ll} p_{\text {CMS}} = 5\times 10^{-3}, \\ \sigma _{\text {CMS}} = 2.6, \end{array} \quad \begin{array}{ll} &{}p_{\text {comb}}= 1 \times 10^{-6}, \\ &{}\sigma _{\text {comb}}= 4.7, \end{array} \end{aligned}$$

where $p_{\text {ATLAS, CMS, comb}}$ $(\sigma _{\text {ATLAS, CMS, comb}})$ correspond to the local *p* value (confidence level) of ATLAS, CMS, and their naive combination.^1^

The local results quoted in Eq. (1.1) are quite significant and captured the attention of the high energy community. Interpreting them naively, one would be lead to one of the following conclusions:

(i) this excess is a result of a rare statistical fluctuation;
(ii) this excess implies a discovery of non-Standard Model dynamics.
  As both conclusions are quite extraordinary (certainly the second one), they motivate an investigation into their robustness. In particular, we raise a third option, to be considered in conjunction with (i), namely, we ask how unlikely is the possibility that
(iii) the significance of the excess is overestimated due to underestimating fake-based backgrounds.

With the inclusion of more data in the analyses the excess eventually vanished [3, 4], ruling out the new physics hypothesis (ii). However, the possibility of claim (iii) remains unclear, affecting all analyses which rely on a precise knowledge of the photon faking background and use the same techniques to estimate it.

While our conclusion is independent of the 750 GeV resonance we use it as an example case to scrutinize the hypothesis of the underestimated background and its implications. First, the main rationale behind our hypothesis is presented in Sect. 2, followed by a detailed description of our approach to background estimation (Sect. 3) and the statistical treatment of the data (Sect. 4). The comparison with the full 2016 data set is presented in Sect. 5. Our main conclusions are summarized in Sect. 6. For other relevant work, see Refs. [5, 6].

## The rationale

Superficially, the experimental situation related to the diphoton excess was fairly straightforward. The experiments had reported a relatively narrow “bump”, $\Gamma /m\lesssim 6\%\ll 1$. Such a bump implies a rise in the differential distribution while, due to the rapidly falling parton luminosity functions, it is expected that any reasonable background-related distribution should be a monotonically decreasing function of the invariant mass. Consequently, the presence of a non-Standard Model feature seemed to have been indicated by the measurements. While this was qualitatively correct the challenge is to quantify the significance of the excess. To endow the bump with a significance, one needs to control and quantify the background.

The following approaches can be used to constrain the form of the background:

**I.** *Data-driven approach* Assuming $\Gamma /m\ll 1$ and a featureless monotonic background, a robust way to constrain it is through *interpolation* via a two-sided side band analysis. However, this requires one to have enough measured events at invariant masses both below the resonance and above it. In the case of the 750 GeV excess, there were less than 40 events in all of the analyses measured with invariant masses above 850 GeV. Such a small number of events does not allow one to use this method reliably.

**II.** *“First-principle”/Monte-Carlo approach* There is a rather narrow class of observables for which the theory has reached an advanced enough level such that we can fully trust our ability to correctly predict the shape of the background distributions. We believe that the invariant mass distribution of experimentally measured diphoton events does not (yet) belong to this selected class of observables. Namely, the continuous diphoton distribution consist of an admixture of two dominant components: (i) The first is made of two real isolated hard photons. This diphoton distribution is currently known to next-to-next-to-leading order (NNLO) accuracy [7, 8] in perturbative QCD and imposing cuts similar to the ATLAS spin-0 analysis suggests an overall uncertainty of about 5% for the invariant mass distribution [8]. (ii) An additional important background component is due to fakes coming mostly from processes involving a hard photon and a jet that passes the various photon quality and isolation cuts [9]. In addition, depending on these cuts, also the dijet background could play an important role. The prompt photon–jet cross section is currently known at next-to-leading order (NLO) in QCD, and several codes are available to produce the relevant distributions, including JetPhox [10] and PeTeR [11]. In addition, QCD threshold resummation at next-to-next-to-next-to-leading logarithmic (N${}^3$LL) order [12, 13] as well as electroweak Sudakov effects are being included [14], resulting in theory uncertainties of about 10–20% [9, 14, 15]. However, a comparison with the 8 TeV ATLAS measurement [9] shows that at low photon $p_T\sim 50\,$GeV the data exhibits some level of deviations from the theoretical predictions (a larger uncertainty is found for the invariant mass distribution; see [16]). In addition, it is important to note that the fake rate strongly depends on the quark/gluon “flavor” of the tagged jet (for some discussion of jet flavor definitions, see [17–20]): intuitively one can understand the difference through the quark and gluon fragmentation functions to pions. At large *x*, as required to be able to pass photon isolation criteria, gluon fragmentation to few pions is much more suppressed (see e.g. Chapter 20 in Ref. [21]). Accordingly, a dedicated ATLAS study [22] found that there is a probability of about $1:2\times 10^3$ for a quark jet to fake a photon, and only $1:2\times 10^4$ for a gluon jet to fake a photon, for jets with $E_T>40\,$GeV. Applying this to the photon–jet background, we also note that subleading jets might become an important source of fakes if the leading jet is predominantly gluon-initiated.

In order to theoretically predict the purity of the diphoton mass distribution, an appropriate admixture of the diphoton and the photon–jet(s) components needs to be constructed [23]. Furthermore, for the latter component, one is required to convolve the photon–jet distribution with the relevant fragmentation functions or at least tag the flavor of the jet(s). It is also important to note that the purity is a highly phase space dependent quantity. Not only does it depend on the ratio of the differential jet–photon and photon–photon production but also on the jet-to-photon fake rate. The fake rate may exhibit a strong dependence on the differential quantities such as $p_T$ and (pseudo)rapidity $\eta$. For instance, as discussed below, in the CMS analyses purity is estimated to be better than 90% in the (central-central) EBEB event category but only better than 80% in the (forward-central) EBEE one. Both experiments consider the purity in an inclusive way. However, in the relevant kinematical region the data is not sufficient to constrain possibly large deviations from the inclusive purity estimation (see Fig. 4).

**III.** *Functional-fit approach* Given the present practical limitations of the methods **I** and **II**, one is lead to a more phenomenological approach in which the background estimate is obtained by fitting an universal function to control regions in the data and then *extrapolating* into the signal regions using the fitted functional form. This allows one to predict the background at relatively high invariant masses in a straightforward manner. Consequently, both experiments are essentially following this approach in most of their analyses,^2^ although the functional forms used by ATLAS in the spin-0 analysis and by CMS are slightly different. Thus, the significance of the excess is mostly determined by comparing measured events to a background estimate predicted by a fitting function.

While method **III** is very transparent and makes the search for bumps easy to analyze, it is also rather susceptible to systematic effects, in particular a lack of understanding of the physics modifying the tails of the distributions, as we argue below. The fitting functions used by ATLAS and CMS are well suited for describing rapidly falling distributions and are fitted to the available data. With the amount of data in the 2015 data sets, the differentially measured number of events is abundant in the low invariant mass region and is spare in the high mass region. The extraction of the functions’ parameters is thus dominantly controlled by the low $m_{\gamma \gamma }$ region and hardly affected by modifications of the invariant mass distribution at diphoton masses of above roughly 500 GeV. However, the significance of the excess with respect to the fitting function is very much affected by such deformations. As it is hard to directly test or predict the correct form of the diphoton mass distribution, this raises the following questions:

${\mathcal {I}}$. Is the experimental signal over background estimation robust against the presence of deviations from the fitting function predictions at large invariant masses?

$\mathcal{II}$. If this is not the case, can one produce smoking-gun predictions to show that indeed the significance of the excess is being overestimated?

Let us first focus on point $\mathcal{I}$. To examine the sensitivity of the significance of the excess to the variation of the tails of the distributions. We consider a family of background shapes that are formed by an admixture of the diphoton and photon–jet distributions. We keep the overall inclusive purity of the samples at 90 and 80%, respectively, in accordance with the measured data at low invariant masses. More specifically, we use two classes of deformations. The first is derived from a modification of the photon–jet spectrum due to NLO and showering effects combined with an increased fake rate for larger transverse momenta and pseudo-rapidities of the jets.

We then consider a simpler ansatz where we allow the distribution of the $p p \rightarrow \gamma j$ component to be reweighted at invariant masses above 500 GeV such that the purity of events with large invariant masses is reduced leading to a controlled deviation from the functional fit. In the following section we provide a detailed description of our approach. We also provide some tests of our procedure to check that our method complies with public data (below and above the resonance region) and is passing the relevant statistical tests. We then report how the significance is affected by the amount of rescaling of the distributions of fakes. Finally we can use our ansatz to address item $\mathcal{II}$ and provide smoking guns to test our hypothesis on overestimating the excess significance. With the full statistics of the 2016 data sets at hand it would be fairly easy to eliminate our hypothesis.

## Reducible and irreducible backgrounds

The main background to the diphoton signal is the irreducible $p p \rightarrow \gamma \gamma$ background. We consider in the following: ATLAS spin-0 and spin-2, and CMS $13\,\,\text {TeV}$ EBEB and EBEE categories with magnets on. We generate the diphoton invariant mass spectrum at NNLO with MCFM version 8.0 [8, 24–27] applying the cuts as described in the respective analyses; see Table 1. The main contribution to the reducible background is the $p p \rightarrow \gamma j$ production where the hard jet is a quark jet that is wrongly reconstructed as a photon. We generate this background at leading order (LO) with MadGraph5 version 5.2 [28]. We note that at LO the $p p \rightarrow \gamma j$ sample is dominated by quark jets, which, as already mentioned, lead to a much larger fake rate than gluon jets.

**Table 1:** Cuts of the analyses where the subscript refers to the hardest and second hardest photon candidate, the cross section of the $p p \rightarrow \gamma \gamma$ sample passing these cuts (calculated at NNLO with MCFM) and of the $p p \rightarrow \gamma j$ sample at hadron level (before applying any photon mistag rate), calculated at NLO with MadGraph5_aMC@NLO, showered with Pythia [29] and the jets clustered with anti-$k_T$, $R=0.4$ algorithm using FastJet [30]. In the last line the ratios of the two distributions in the invariant mass region above 500 GeV are given

Analysis	ATLAS spin-0	ATLAS spin-2	CMS EBEB	CMS EBEE
$m_{\gamma \gamma }$	${>}150\,\,\text {GeV}$	${>}200\,\,\text {GeV}$	${>}230\,\,\text {GeV}$	${>}330\,\,\text {GeV}$
$p_{T,1}$	${>}0.4\,m_{\gamma \gamma }$	${>}55\,\,\text {GeV}$	${>}75\,\,\text {GeV}$	${>}75\,\,\text {GeV}$
$p_{T,2}$	${>}0.3\,m_{\gamma \gamma }$	${>}55\,\,\text {GeV}$	${>}75\,\,\text {GeV}$	${>}75\,\,\text {GeV}$
$\left| \eta _1\right|$	${<}2.37$	${<}2.37$	${<}1.44$	${<}1.44$
$\left| \eta _2\right|$	${<}2.37$	${<}2.37$	${<}1.44$	$1.57<\eta _2<2.5$
$\left| \eta \right|$ excluded	$1.37<\eta _{1,2}<1.52$	$1.37<\eta _{1,2}<1.52$	n.a.	n.a.
$\sigma _{\gamma \gamma }$ [pb] (NNLO)	2.7	1.9	0.52	0.23
$\sigma _{\gamma j}$ [pb] (NLO)	1400	1000	250	130
$\left. {\sigma _{\gamma j}}{}/{\sigma _{\gamma \gamma }}{}\right| _{m>500\,\,\text {GeV}_{}}$	510	670	470	640

The reconstructed diphoton distribution is a mixture of $p p \rightarrow \gamma \gamma$ and $p p \rightarrow \gamma j$ invariant mass distributions. Let us define a short-hand notation for the normalized invariant mass distribution

3.1 $$\begin{aligned} w_{\gamma X}\equiv \frac{1}{\sigma _{\gamma X}} \times \frac{\text {d}\sigma _{\gamma X}}{{\text {d} m_{\gamma X}}}, \end{aligned}$$

with $X=\gamma ,j$. The mixed distribution $w_\text {mix}$ is a function of the normalization $N_\text {mix}$ and a parameter $\mathcal {R}$, that controls the shape modification of $w_{\gamma j}$ and will be defined Eq. (3.4). We write $w_\text {mix}$ as

3.2 $$\begin{aligned} w_\text {mix}(N_{\text {mix}}) =N_{\text {mix}} \left[ \mathcal {P}w_{\gamma \gamma }+(1-\mathcal {P})w_{\gamma j} \right] , \end{aligned}$$

where $\mathcal {P}$ is the inclusive purity of the sample. We set $\mathcal {P}=90\%$ (80%) for the ATLAS and CMS EBEB (EBEE) analyses, which is within the reported error bands. We will assume, that $w_{\gamma \gamma }$ is obtained by normalizing the MCFM diphoton invariant mass distribution. As for $w_{\gamma j}$, following the rationale described in Sect. 2, we modify things in two different ways as we now describe in detail.

### QCD and jet-fake dependence of the diphoton shape

**Fig. 1:**
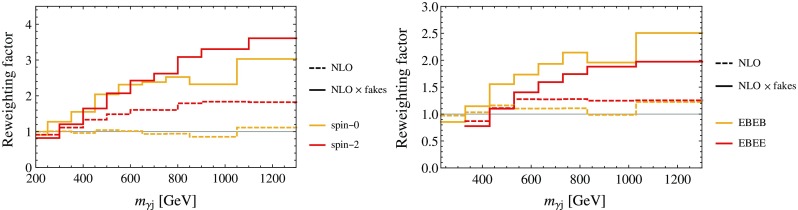
Reweighting factor for $w_{\gamma j}$ with respect to the LO Monte Carlo distribution as a function of the invariant mass for ATLAS (*left*) and CMS (*right*). The *dashed lines* show the reweighting factor to modify the LO parton distribution $w_{\gamma j}^\text {MG}$ to the NLO shape, including the effects of hadronization. The *solid lines* include in addition the reweighting due to a phase space dependent fake rate

First, we calculate a photon–jet mass dependent K-factor using MadGraph5_aMC@NLO, showered and hadronized with Pythia [29] and jets clustered with an anti-$k_T$, $R=0.4$ algorithm [31] using FastJet [30]. We note that in the NLO distribution we only consider the hardest jet of the event and we do not record its flavor. This step may be potentially improved by the use of an IR-safe jet flavor definition, see [17–20]. Next, in order to model the dependence of the fake rate on the pseudo rapidity and the transverse momentum we use the following simplified ansatz for the jet rejection $r(p_T,\eta )$:

3.3 $$\begin{aligned} r(p_T,\eta ) = {\text {max}} \left\{ \frac{r_0}{1+ p_T/p_T^0 + \eta /\eta ^0}, r_{\text {min}}\right\} \,, \end{aligned}$$

where the functional form is motivated by the kinematical dependence of the jet-rejection rates as estimated by ATLAS [22] and the parameter values $p_T^0 = 30$ GeV, $\eta ^0 = 4$ are chosen to reproduce the rejection rate ratios between the lowest and highest lying $\eta$ and $p_T$ bins within uncertainties. Finally, $r_0/r_{\text {min}}$ is fairly uncertain as estimates of rejection rates at very high $p_T$ and $\eta$ are not publicly available, but reproducing experimental purity estimates in the forward region [2] leads to values in the wide range $r_0/r_{\text {min}} \in [3,12]$. The resulting reweighting factors compared to the LO partonic $m_{\gamma j}$ distribution obtained from MadGraph5, $w_{\gamma j}^{\text {MG}}$, at both steps applied successively ($w_{\gamma j}^{\text {NLO}}/w_{\gamma j}^{\text {MG}}$ and $w_{\gamma j}^{\text {NLO}\,\times \,\text {fakes}}/w_{\gamma j}^{\text {MG}}$) are shown in Fig. 1. We observe that with our choice of fake rate parameters, the largest reweighting factors close to 3 are obtained above $m_{\gamma j} > 800$ GeV for the ATLAS spin-2 cuts. However, all experimental categories are affected by a reweighting factor which is a combination of NLO, hadronization and faking effects, and which increases with the photon–jet invariant mass until it saturates at some point. This suggests a simple functional form for the effective photon–jet spectrum deformation which we discuss next.

**Fig. 2:**
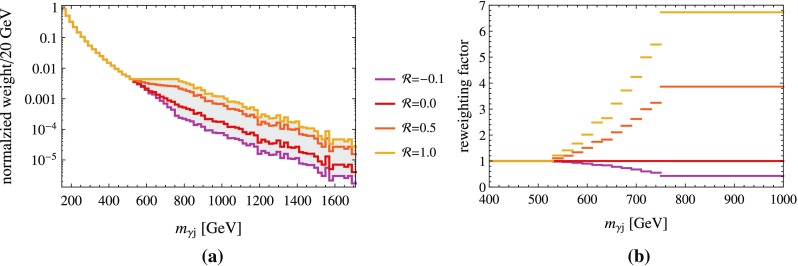
*Left* Normalized invariant mass distributions after the ATLAS spin-0 cuts for the $p p \rightarrow \gamma j$ background with several choices for the interpolating parameter $\mathcal {R}$. *Right* Corresponding reweighting factor

### Effective shape deformation

In our effective ansatz for the deformation of the photon–jet spectrum, we focus on the invariant mass region above $m_{\gamma \gamma } > 500$ GeV below which the experiments have sufficient statistics to control the mass distributions and calibrate their analyses and choose a simple, linear form,

3.4 $$\begin{aligned}&w_{\gamma j}(\mathcal {R})=\frac{w_{\gamma j}^{\text {MG}}}{N(\mathcal {R})}\nonumber \\&\quad \left[ 1+\mathcal {R} \times {\left\{ \begin{array}{ll} 0 &{} m_{\gamma j}< 500\,\,\text {GeV}\\ \frac{\left. w_{\gamma j}^\text {MG}\right| _{m_{\gamma j}=500\,\,\text {GeV}}}{w_{\gamma j}^\text {MG}}-1 &{} 500\,\,\text {GeV}\le m_{\gamma j} \le 760\,\,\text {GeV}\\ \frac{\left. w_{\gamma j}^\text {MG}\right| _{m_{\gamma j}=500\,\,\text {GeV}}}{\left. w_{\gamma j}^\text {MG}\right| _{m_{\gamma j}=760\,\,\text {GeV}}} -1 &{} 760\,\,\text {GeV}< m_{\gamma j} \end{array}\right. } \right] , \end{aligned}$$

to roughly account for an overall kinematic dependence of the fake rate, hadronization and higher order effects $N(\mathcal {R})$ is an $\mathcal {R}$-dependent normalization factor. We define $w_{\gamma j}(\mathcal R)$ such that $w_{\gamma j}(\mathcal {R}=0)$ corresponds to the partonic LO distribution. Choosing $\mathcal {R}=1$, the shape of $w_{\gamma j}$ is unmodified for $m_{\gamma j}<500\,\,\text {GeV}$, then flat up to $m_{\gamma j}=760\,\,\text {GeV}$, just above the observed peak of the apparent excess, and finally it is rescaled by the ratio of the differential cross sections at $m_{\gamma j}=500\,\,\text {GeV}$ and $m_{\gamma j}=760\,\,\text {GeV}$ for $m_{\gamma j}>760\,\,\text {GeV}$. The $\mathcal {R}$-dependence of $N(\mathcal {R})$ is chosen such that the integral over $w_{\gamma j}$ is always 1, independent of the value of $\mathcal {R}$. For the ATLAS spin-2 and the two CMS analyses, the intervals in the above equation are shifted by $10\,\,\text {GeV}$ to larger values due to the different binning in these searches.

In the left panel of Fig. 2, we show the normalized differential $p p \rightarrow \gamma j$ cross section for the ATLAS spin-0 analysis as a function of the invariant mass for several choices of $\mathcal {R}$. In the right panel, the $\mathcal {R}$-dependent reweighting factor of Eq. (3.4) is shown. Since the spin-0 analysis applies the strongest cuts on the transverse momenta of the photon candidates ($0.4\,m_{\gamma \gamma }$ and $0.3\,m_{\gamma \gamma }$, respectively) its distribution is the steepest. Thus the reweighting factor of this analysis is the largest being almost 7 above 770 GeV. The maximal reweighting factors for the other analyses are just above 6. We verified that increasing the flat region by 20 GeV has only a small impact on the reported results.

**Fig. 3:**
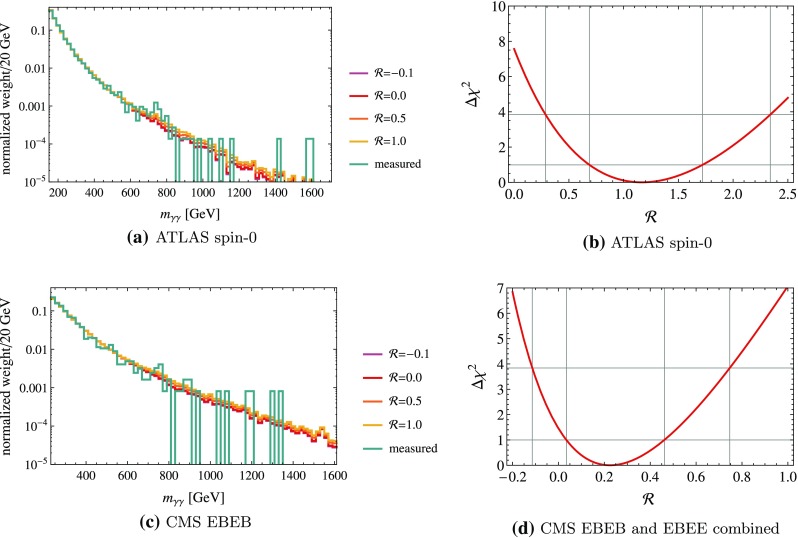
*Top left* Combined distributions $w_\text {mix}$ for the ATLAS spin-0 cuts, all with an overall purity of 90%. The distribution obtained from the 2015 data sets is shown in *blue*. *Bottom left* Corresponding distribution for the CMS EBEB analysis. *Top right* $\Delta \chi ^2$ of fit to the ATLAS spin-0 distribution as a function of $\mathcal {R}$. The 1- and 2-$\sigma$ regions are indicated by the *thin lines*. *Bottom right* corresponding *plot* for the combined CMS EBEB and EBEE analyses

In Fig. 3a, c, we show the resulting invariant mass distributions $w_{\text {mix}}$ for the ATLAS spin-0 and CMS EBEB analysis, respectively, on top of the normalized distribution as measured in the 2015 data set.

In addition to the average purity of the full sample, ATLAS and CMS try to estimate the purity as a function of the diphoton invariant mass. This local purity is given by

**Fig. 4:**
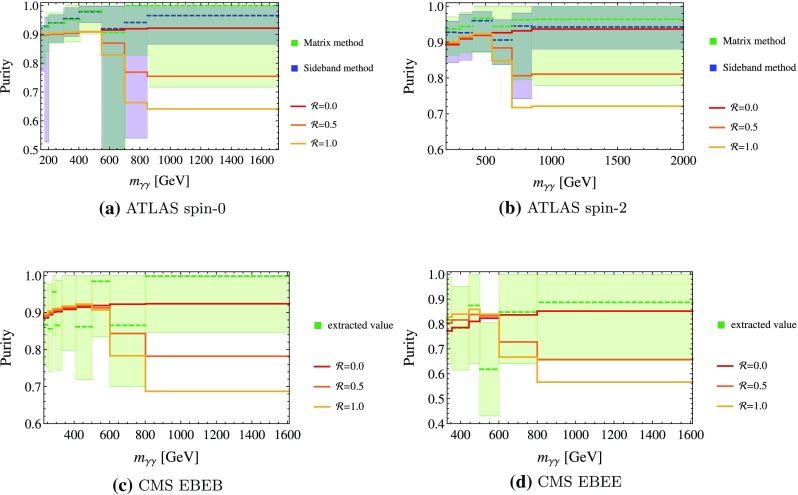
Purity of the combined distribution as a function of $m_{\gamma \gamma }$ for several choices of $\mathcal {R}$. The *dashed lines* show the central value for the purity as determined by the experiments and shaded the areas show the corresponding error

3.5 $$\begin{aligned} \mathcal {P}^i=\frac{\mathcal {P} w^i_{\gamma \gamma }}{\mathcal {P} w^i_{\gamma \gamma } + (1-\mathcal {P}) w^i_{\gamma j}} \end{aligned}$$

for the *i*th bin. It can deviate significantly from the average purity $\mathcal {P}$ of the full sample. In Fig. 4, we show the binned purities for the mixed samples with several choices of $\mathcal {R}$ compared to the purity determined by ATLAS with the $2\times 2$ sideband [32] and the matrix method [33] and by CMS with a method described in [34], respectively. We choose the same binning of the purity as is used in the respective analysis.

While the local purity is within the error band in most of the considered mass range (even for $\mathcal {R}=1$), it does decrease for large invariant masses and our ansatz predicts a deviation from the experimental value. Given the low statistics in this range, we consider this as a way to falsify our proposal in the future rather than a contradiction with the currently available data.

## Statistical treatment

The experimental analyses estimate the background shape by fitting a function *f*(*x*) with $x=m_{\gamma \gamma }/\sqrt{s}$ to the measured data. In the ATLAS spin-0 analysis, the following ansatz is used:

4.1 $$\begin{aligned} f(x)=N\left( 1-x^{1/3}\right) ^b x^{\sum _{j=0}^k a_j \left( \log x\right) ^j} \end{aligned}$$

where $k=0$ was chosen. For the CMS analyses as well as the ATLAS spin-2, we use

4.2 $$\begin{aligned} f(x)=Nx^{a+b\log x}. \end{aligned}$$

Note that the ATLAS spin-2 analysis uses a mixture of Monte Carlo (for the $p p \rightarrow \gamma \gamma$ background) and data-driven distributions (for the $p p \rightarrow \gamma j$ and $p p \rightarrow jj$ background), leading to similar results as the fit function approach. In the data-driven method, the shape of the different backgrounds is obtained by extracting the corresponding events from control samples and fitting their distribution with a function. The relative contribution to the observed $pp \rightarrow \gamma \gamma$ sample is extracted from the data between $200\,\,\text {GeV}< m_{\gamma \gamma }<500\,\,\text {GeV}$. For more details on this method see [1]. Given the small statistics in the large invariant mass bins this approach roughly corresponds to our LO MG distribution.

In order to see how the significance of the 750 GeV excess changes with our ansatz, we fit the distribution $w_{\text {mix}}$, defined in Eq. (3.2), once with $w_{\gamma j}^{\text {MG}}$ corresponding to $\mathcal R=0$, and then with $w_{\gamma j}^{}$ as estimated at NLO with showering and hadronization, including fakes and finally with $\mathcal {R}$ as a free fit parameter (as well as the appropriate fit function *f*(*x*)) to the measured data. As an additional template, one could extend the fit function *f*(*x*) by a modification similar to the one described in Eq. (3.4), which we will, however, not do for the sake of simplicity. The fits are performed with two methods, which yield similar results.

Firstly, we maximize the likelihood

4.3 $$\begin{aligned} L=\prod _{i=1}^{N_\text {bins}} P_{N_\text {e}^i}(N_\text {m}^i) \end{aligned}$$

where the product goes over all bins and $P_{N_\text {e}}(N_\text {m})$ is the Poisson probability to measure $N_\text {m}$ events when $N_\text {e}$ events are expected.

Secondly, we minimize

4.4 $$\begin{aligned} \chi ^2=\sum _{i=1}^{N_\text {bins}} (N_\text {m}^i-N_\text {e}^i)^2/N_\text {e}^i \end{aligned}$$

where we rebin the data such that each bin contains at least 10 events in order for the $\chi ^2$ distribution to provide a reasonable description of the statistical uncertainties; see e.g. [35]. In both fit methods, the overflow of the experimental histograms is treated as one single bin. The best-fit parameters determine the number of expected events $N_\text {e}$ in the signal region (SR).

Since we are mostly interested in the local significance of the 750 GeV excess, the SR is chosen by eye from the measured distribution with the aim to capture the excess. We obtain a *p*-value by comparing the number of measured events in the SR $N_\text {m}$ with $N_\text {e}$ (more precisely: calculating the Poisson probability to measure at least $N_\text {m}$ events):

4.5 $$\begin{aligned} p=\sum \limits _{n=N_\text {m}}^\infty \frac{N_\text {e}^n}{n!} e^{-N_\text {e}}. \end{aligned}$$

Clearly this simple approach which does not use any signal modeling and relies on a discrete width and position of the SR is far from perfect. Consequently, it is not surprising that the obtained significances of the excess are smaller than the ones reported by the experiments, even when we use the same fit functions. Instead of focusing on absolute values one should therefore rather consider the *reduction* of the significance that results from modifying the background. The results of the fits are shown in Table 2.

**Table 2:** Results of the fits to the data of all four analyses. In the first block from the top the signal region is defined and the number of measured events in this region $N_m$ is given. The results of a likelihood- and a $\chi ^2$ fit of the fit function (value of the maximal likelihood and minimal $\chi ^2$, respectively, and the local significance of the $750\,\,\text {GeV}$ excess) are given in the second block. Finally, the third block contains the results of a likelihood and $\chi ^2$ fit of the background distributions described in Sect. 3 to the data. When $\mathcal {R}$ was fitted its best-fit value and the corresponding local significance of the excess are given, otherwise just the significance. In the last line the result of the F-test, testing whether $\mathcal {R}$ should be used as fit parameter, is given. For the minimized $\chi ^2$ the parameter *n* is the difference of number of bins and fit parameters. The errors indicate the 1-$\sigma$ interval of the systematic uncertainty of the fit. Note that the results of the spin-0 analysis with $3.2\,\text {fb}^{-1}$ are based on the analysis with looser photon identification as described in [1]

Analysis	ATLAS spin-0		ATLAS spin-2	CMS EBEB		CMS EBEE	
Measurement							
SR	730–770 GeV	720–760 GeV	720–780 GeV	710–770 GeV		710–770 GeV	
$\int \mathcal {L}\,\text {d}t$	$3.2\,\text {fb}^{-1}$	$15.4\,\text {fb}^{-1}$	$3.2\,\text {fb}^{-1}$	$2.7\,\text {fb}^{-1}$	$12.9\,\text {fb}^{-1}$	$2.7\,\text {fb}^{-1}$	$12.9\,\text {fb}^{-1}$
$N_m$	15	33	40	12	24	21	53
Fitfunction							
$-2\log L$	270	–	330	200	–	200	–
$\sigma$	3.4	–	2.9	1.9	–	1.7	–
$\chi ^2/n$	1.6	0.75	1.2	0.80	1.0	1.2	0.98
$\sigma$	$3.1_{-0.2}^{+ 0.2}$	$1.2^{+0.2}_{-0.2}$	$2.9_{-0.3}^{+ 0.3}$	$1.8_{-0.2}^{+ 0.3}$	$-1.5^{+0.2}_{-0.3}$	$1.6_{-0.3}^{+ 0.3}$	$-1.3^{+0.2}_{-0.2}$
Distribution							
$$\mathcal {R}$$							
$-2\log L$	270	360	330	210	310	210	300
$\mathcal {R}$	0.86	0.11	0.97	−0.048	−0.7	0.24	−0.12
$\sigma$	2.2	0.0	2.0	1.5	−1.2	1.4	−0.23
MG							
$\chi ^2/n$	1.5	0.76	1.3	0.78	1.6	1.1	1.1
$\sigma$	$3.0_{-0.0}^{+ 0.0}$	$0.2^{+0.0}_{-0.0}$	$3.4_{-0.1}^{+ 0.1}$	$1.4_{-0.1}^{+0.1}$	$-2.6^{+0.1}_{-0.1}$	$1.9_{-0.2}^{+ 0.2}$	$-0.86^{+0.14}_{-0.14}$
NLO							
$\chi ^2/n$	1.5	0.76	1.2	0.80	1.6	1.2	1.0
$\sigma$	$3.0_{-0.0}^{+0.0}$	$0.3^{+0.0}_{-0.0}$	$3.2_{-0.1}^{+0.1}$	$1.4_{-0.1}^{+0.1}$	$-2.6^{+0.1}_{-0.1}$	$1.7_{-0.2}^{+0.2}$	$-1.2^{+0.1}_{-0.1}$
NLO$\times$fakes							
$\chi ^2/n$	1.5	1.4	1.1	0.92	2.0	1.2	1.2
$\sigma$	$2.7_{-0.0}^{+0.0}$	$-0.3^{+0.0}_{-0.0}$	$2.9_{-0.1}^{+0.1}$	$1.2_{-0.1}^{+0.1}$	$-3.0^{+0.1}_{-0.1}$	$1.4_{-0.2}^{+0.2}$	$-1.7^{+0.1}_{-0.1}$
$$\mathcal {R}$$							
$\chi ^2/n$	1.2	0.75	1.0	0.81	1.3	1.1	1.1
$\mathcal {R}$	$1.2_{-0.5}^{+ 0.6}$	$0.2^{+0.2}_{-0.2}$	$1.1_{-0.4}^{+ 0.4}$	$-0.15_{-0.39}^{+ 0.51}$	$-0.6^{+0.2}_{-0.2}$	$0.30_{-0.22}^{+ 0.29}$	$-0.091^{+0.084}_{-0.074}$
$\sigma$	$2.0_{-0.4}^{+ 0.4}$	$-0.2^{+0.4}_{-0.4}$	$1.9_{-0.5}^{+ 0.5}$	$1.5_{-0.4}^{+ 0.4}$	$-1.3^{+0.4}_{-0.4}$	$1.2_{-0.4}^{+ 0.4}$	$-0.40^{+0.42}_{-0.42}$
$p_\text {F-test}$	0.021	0.20	0.0027	0.73	0.014	0.19	0.30

Finally, an *F* test is performed to determine if the generalization of our mixed distribution with $w_{\gamma j}^{\text {MG}}$ to the one with $w_{\gamma j}(\mathcal {R})$ given in Eq. (3.4) is needed to describe the data. This test investigates the improvement of a fit when the fit function is extended by an additional parameter. For this purpose, a test statistic

4.6 $$\begin{aligned} F=\frac{(\chi ^2_1-\chi ^2_2)/(n_1-n_2)}{\chi ^2_2/n_2} \end{aligned}$$

is calculated, where $\chi ^2_{1,2}$ are the minimized $\chi ^2$ of the two fit functions, $n_{1,2}$ are the numbers of bins (27 for the ATLAS spin-0) minus the number of input parameters (3 vs. 4 for the fitting function and 1 vs. 2 for our distribution), and the subscripts refer to the two fit functions with 2 signifying the extended function. Eventually the *p*-value is determined as

4.7 $$\begin{aligned} p_\text {F-test}=\int _F^{\infty } \digamma (x; n_1-n_2,n_2)\text {d}x, \end{aligned}$$

with $\digamma$ being the Fisher distribution. An additional fit parameter is warranted if $p_\text {F-test}<5\%$; see [1].

We find that, for the ATLAS searches, the F-test suggests that $\mathcal {R}$ should be included as a fitting parameter. The probability of an accidental improvement due to $\mathcal {R}>0$ is only $2.1\%$ (ATLAS spin-0) and $0.27\%$ (ATLAS spin-2). On the other hand, the CMS categories do not prefer a significant non-zero $\mathcal R$; see Table 2. Furthermore, as a consistency check, we apply the F-test on the ATLAS fitting function for spin-0, Eq. (4.1), and find that adding a $k=1$ component to the function does not pass the test. Hence, as mentioned in [1] only the leading term of the function with $k=0$ is retained. The above in conjunction with the results collected in Table 2 suggest that it is possible that the basis of functions used in Eq. (4.1) is not sufficient to accommodate the deformation of the distribution proposed by us (or at least not the first term in the functional form).

In the $\chi ^2$ fit of the constructed distribution with $w_{\gamma j}^{MG}$ we find similar results for the local significance as with the $\chi ^2$ fit of the functional approach. However, in particular in the two ATLAS analyses, using $\mathcal {R}$ as an additional fit parameter reduces the local significance of the 750 GeV excess by 1–1.5 units. The fact that the reduction is stronger in the spin-2 analysis corroborates our working assumption that the background description deteriorates in the forward region. This is further supported by the observation that in the CMS EBEB analysis, which collects only events with both photon candidates in the central region, no reduction in significance is observed and the best-fit value for $\mathcal {R}$ is even slightly negative. Only in the CMS EBEE analysis where one photon candidate is in the forward region the significance is reduced by fitting $\mathcal {R}$, albeit less than in the ATLAS analyses.

**Fig. 5:**
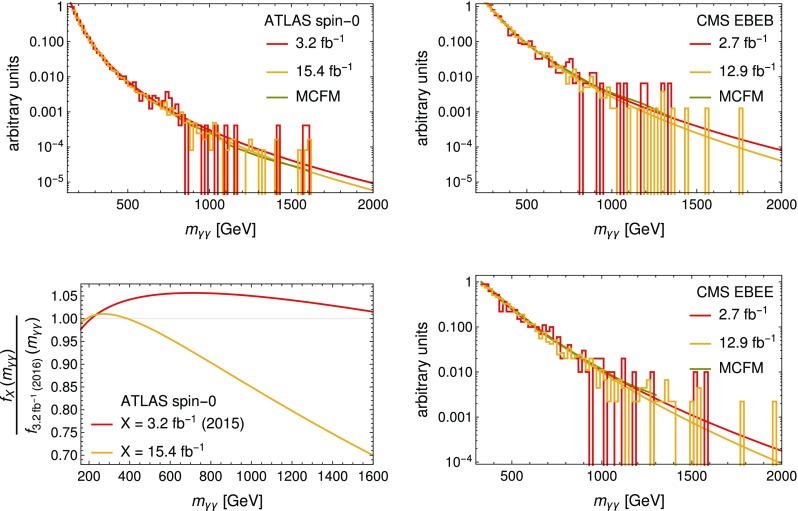
*Upper plots* and *lower right plot* Comparison of the measured and fitted distributions with the small and the full data sets. In addition, the pure digamma spectrum as obtained from MCFM is shown. In the *upper left plot* the comparison is between the smaller Moriond 2016 data set with the old photon isolation method and the full ICHEP 2016 data set. The distributions and functions are normalized to have the same value at the low $m_{\gamma \gamma }$ end of the histograms.The *lower left plot* shows the ratios of the normalized fit functions $f_X$ fitted to the ATLAS spin-0 data set *X* with the year 2015 (2016) in the *brackets* indicating the old Moriond (updated ICHEP) photon isolation criteria

**Fig. 6:**
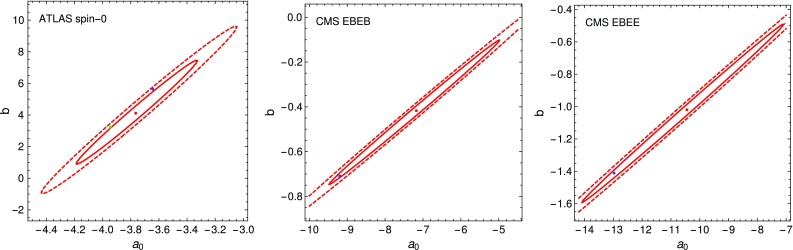
Best-fit point of the $\chi ^2$ fit of the appropriate function to the Moriond 2016 data set in red with the 1- and 2-$\sigma$ contours. The best-fit point for the fit to the full ICHEP 2016 data set is shown in *blue* and in the *left plot* the best-fit point for the ATLAS spin-0 $3.2\,\text {fb}^{-1}$ data set with the new photon isolation is shown in *green*

**Fig. 7:**
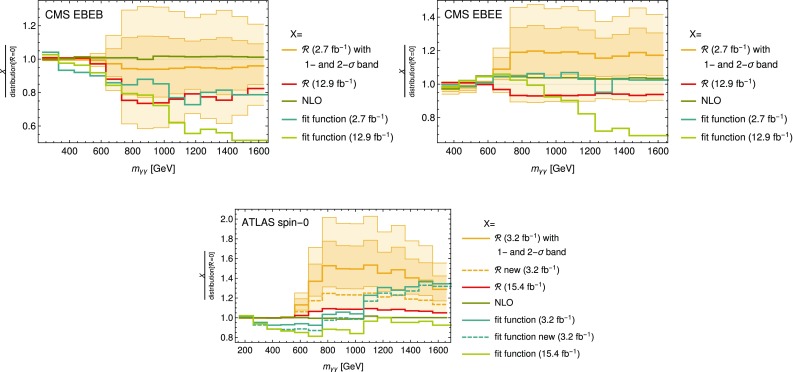
*Plot* of the normalized distributions with several choices of $\mathcal {R}$ and normalized fit functions to the old and new data sets, all divided by the distribution for $\mathcal {R}=0$. In the *plot* for the ATLAS spin-0 analysis the *dashed lines* show the results obtained using the old data set with the new photon isolation criteria

Since $\mathcal {R}>0$ flattens the distribution one might worry that the reduction in the local significance is obtained by overshooting the measured distribution in the high invariant mass region. By verifying that both the minimal $\chi ^2$ and the maximal likelihood hardly change between the functional and the distribution fit we show that this is not the case.

As a final exercise, we try to obtain a “combined” significance from the analyses of the 2015 data set. Clearly a proper statistical combination cannot be done, since we neglect correlations between the various analyses and also fit for a single universal value of $\mathcal {R}$. Realistically, $\mathcal R$ is expected to be somewhat different for the different analyses since they cover different regions of phase space. Nevertheless, since the naive combination in Eq. (1.1) suffers from similar issues we set them aside and proceed as follows. We sum the $\chi ^2$ of the analyses included in the combination and fit for a common $\mathcal {R}$ while keeping the normalizations as separate variables. By combining the two CMS analyses we obtain $\sigma =2.4$ (1.9) for $w_{\gamma j}^{\text {MG}}$ (with $w_{\gamma j}(\mathcal {R})$, best-fit $\mathcal {R}=0.22$) and $\sigma =1.9$ with $w_{\gamma j}^{\text {NLO}\times \text {fakes}}$. A combination of the ATLAS analyses is impossible since they are not independent. However, we can combine each of them with the two CMS analyses and obtain for ATLAS spin-0 combined with CMS $\sigma =3.6$ (2.6 with $w_{\gamma j}(\mathcal {R})$, best-fit $\mathcal {R}=0.46$; 3.1 with $w_{\gamma j}^{\text {NLO}\times \text {fakes}}$) and for ATLAS spin-2 combined with CMS $\sigma =4.2$ (2.8 with $w_{\gamma j}(\mathcal {R})$, best-fit $\mathcal {R}=0.53$; 3.4 with $w_{\gamma j}^{\text {NLO}\times \text {fakes}}$), where the significance numbers before the brackets are obtained for $w_{\gamma j}^{\text {MG}}$.

## The new energy frontier: searches beyond 1 TeV

Around ICHEP 2016, ATLAS and CMS updated their analyses, now based on 15.4 $\text {fb}^{-1}$ and 12.9 $\text {fb}^{-1}$, respectively. In the updated ATLAS spin-0 analysis [3] and the CMS EBEB and EBEE analyses [4] the large excess around $750\,\,\text {GeV}$ vanished and no other significant excesses were found. An update of the ATLAS spin-2 analysis has not been presented. While CMS processed the data exactly as before, ATLAS made some adjustments, perhaps most importantly, using a tighter photon isolation. We repeat the fits and the statistical treatment of the reported results with the same methods as described above and report the results for the larger data set in Table 2. Note that there is a downwards fluctuation in the signal region in the full CMS data set which even leads to a slightly negative significance.

Comparing the new fit functions to the ones based on the previous small data sets presented at Moriond 2016 we find a steeper functional fit in all three analyses; see Fig. 5. While the new best-fit parameters are within one standard deviation for the two CMS fits, the ones for the ATLAS fit deviate by almost two standard deviations after marginalizing over the normalization; see Fig. 6. This might, however, be an effect of the changed photon isolation as the fit to the $3.2\,\text {fb}^{-1}$ data set with the updated photon identification also deviates by more than one standard deviation from the previous best-fit point. A better understanding of the effect of the fake photons could be obtained by investigating the result of changing the isolation criteria with the full $15.4\,\text {fb}^{-1}$ data set. The tighter isolation criteria are also reflected in the better agreement between the fitted distributions and the MCFM generated digamma spectrum.

In order to show the changes in the fits, the ratio of the normalized fit functions for the ATLAS spin-0 analysis is shown in the lower left plot of Fig. 5. A direct comparison of the data and the un-normalized fit functions, even for the fits to the two different $3.2\,\text {fb}^{-1}$ sets, is difficult since the binning of data has changed. The large change in the fit parameters is reflected in the deviation of more than 5% for the comparison of the fits to the two $3.2\,\text {fb}^{-1}$ data sets and the even greater deviation compared with the fit function to the full $15.4\,\text {fb}^{-1}$ data set. While in the previous signal region near 750 GeV the change is of the order of 10% it is greater than 30% near 1.6 TeV. This shows that the actual shape of the digamma spectrum at high invariant masses is hard to predict precisely by an extrapolation and is therefore very much subject to systematic uncertainties.

Finally in Fig. 7 the ratios of several normalized distributions and fit functions to the normalized distribution with $\mathcal {R}=0$ are shown. These include the distributions with the best-fit value for $\mathcal {R}$ based on the Moriond 2016 data set and the ICHEP 2016 data set and also the NLO distributions and the fit functions to the old and new data sets. In the case of the ATLAS spin-0 analysis also the distribution and fit function to the 2015 data set with the new photon isolation is shown. By comparing the curves we find that a sizable systematic uncertainty can be inferred from the differences between the fit functions.

## Conclusions

This paper deals with a problem that often arises in searches for new physics at the energy frontier. In this context the challenge is to look for a new resonance at the upper end of a distribution where only limited knowledge on the SM background is available. As a case study we focus on the 750 GeV anomaly where we examine in particular the implications of the possibility that the excess in the 2015 data set is not only due to a (malicious) statistical fluctuation but also a result of a physical effect. We discuss possible issues with the background: how much photon–jet contamination is still allowed in the region of interest? How could it affect the significance of the excess?

We study these questions using currently available theoretical tools for computing the photon–jet mass distributions and apply them to the small set of publicly available data. However, this approach is limited by our ability to thoroughly disentangle the effects of the $(p_T,\eta )$-dependent jet-fake rate and the theoretical uncertainty of the shape of the photon–jets background. We therefore choose to model these combined effects by an $m_{\gamma j}$ dependent reweighting of the invariant mass distribution, keeping the overall purity within the quoted ranges. We first study a physics-driven reweighting procedure: we convolve a mass dependent K-factor with a rapidity and transverse momentum dependent photon fake rate for the jets. The K-factor is extracted comparing the NLO leading-jet–photon to the LO quark–photon spectrum, and the phase space dependence of the fake rate is estimated from the experimental literature [22]. Both correction factors are approximate, based on incomplete information, and should be taken with a grain of salt. Motivated by this result, we then consider a more phenomenological deformation of the $p p \rightarrow \gamma j$ spectrum. It allows us to study the sensitivity of the significances on a single continuous quantity $\mathcal {R}$ (see Eq. (3.4)) which parametrizes an effective deformation.

To summarize our results for the 750 GeV case study based on the 2015 data we focus on the simpler effective ansatz where we find the following:

- For the ATLAS spin-0 analysis, the significance of the excess can be reduced by $\Delta \sigma \simeq 1.1$ when comparing the fitting function defined in (4.1) with our best fit to the $\mathcal R$-modified distribution. A comparable reduction is found for the ATLAS spin-2 measurement. Here however, it is less straightforward to determine the reduction, since the estimation of the background shape in our ansatz differs from that of the ATLAS analysis, which is not reproducible since the required data is not publicly available. Strictly speaking, $w_{\gamma j}^{\text {MG}}$ does therefore not correspond to the ATLAS approach but is the best approximation we can get. Since, however, ATLAS claims to find comparable results with the corresponding fitting function defined in (4.2), we can reduce the significance with the $\mathcal {R}$-modified distribution with respect to the fitting function by $\Delta \sigma \simeq 1.0$ as well as with respect to the distribution with $w_{\gamma j}^{\text {MG}}$ by $\Delta \sigma \simeq 1.5$.
- The effect is smaller for the CMS 13 TeV analyses with $\Delta \sigma \simeq 0.3-0.4$, depending on the category.
- In a “combined fit” to independent ATLAS and CMS data sets, the significance can be reduced by as much as $\Delta \sigma \approx 1.0 \,(1.4)$ for the ATLAS spin-0 (spin-2) combined with CMS.

The larger preference for an enhanced photon–jet contribution in the spin-2 sample could point to its higher sensitivity to the large rapidity region where jet fakes are more difficult to reject. Finally, an F-test shows that the ATLAS data support using a more complex distribution.

To summarize our results for the 750 GeV case study based on the 2016 data we find the following:

- For the ATLAS spin-0 analysis, we find that the new data prefers $\mathcal {R}$ in the range $0.2\pm 0.2$, eliminating the remaining significance of $1.2\,\sigma$ in the full data set. As for the spin-2 case no data is currently available.
- The updated CMS analyses based on $12.9\,\text {fb}^{-1}$ even have a downwards fluctuation with respect to the fit function near 750 GeV leading to negative significances. Correspondingly the best-fit values for $\mathcal {R}$ are negative and ameliorate the situation.

We emphasize that our simplified ansatz for the effective modification of the photon–jet background with $\mathcal {R}$ is not meant to necessarily represent a new background source nor the exact shape of the background contamination in the signal region. Rather its envelope (corresponding to the shaded area in Fig. 2a) is expected to reflect a possible combination of higher order QCD contributions, fragmentation, isolation and detector effects, which are outside of theoretical control and, in the high invariant mass signal region, also beyond direct experimental probes with currently available data. We further note that a quark flavor tagged dijet sample might provide a high-statistics measurement of the relevant photon fake rates (see for instance [36]).

We have also employed our analysis to compare the difference between the fitting functions used by ATLAS (with the new isolation criteria) given the 2015 and 2016 data sets. The fitting functions where extrapolated to invariant masses beyond the TeV region. In summary we have found that:

- A variation of about 30% in the extrapolated background near $m_{\gamma \gamma }=1.6\,\,\text {TeV}$ is obtained.

To conclude, we have extensively examined the status of LHC diphoton searches. We have compared the analyses performed on both 2015 and 2016 data sets in order to scrutinize the current state of the art measurements for systematic effects. Using our approach we have reevaluated the current experimental sensitivity to beyond standard model physics, especially in the tails of the diphoton invariant mass distributions, beyond the TeV range. We found that the extrapolation of background shapes is subject to sizable uncertainties, potentially affecting the significance of possible future excesses near the edge of the measured distributions. Furthermore, our analysis motivates further Monte Carlo studies of the dominant diphoton backgrounds, based on jet flavor tagging algorithms. Knowledge of whether a jet is of “quark” or “gluon” origin would improve our estimation for the jet-photon faking backgrounds to next-to-leading order QCD accuracy. It is important to note that diphoton-based searches at even larger invariant masses, which are highly motivated, are being performed at present and will continue to be an integral part of the LHC experimental physics program at the high energy frontier.

## Footnotes

1 We do not discuss here the global significance as it strongly depends on the lower value of $m_{\gamma \gamma }$ defined for the search region. ATLAS (CMS) chose it to be about 200 GeV (400 GeV). Furthermore, as discussed below, the region below 500 GeV is dominating the fit to the functional form which is used to estimate the background. Thus, it is not clear whether one should consider this region as a control region or as the region of interest for the search itself.

2 An exception is the ATLAS spin-2 analysis which employs a Monte Carlo approach (**II**) with a data-driven estimate of the photon–jet and jet-jet background; see Sect. 4.
